# Awareness and perceptions of Filipino obstetrician-gynecologists on fertility preservation: a cross-sectional survey

**DOI:** 10.1186/s12905-023-02436-7

**Published:** 2023-05-25

**Authors:** Glaiza S. de Guzman, Eileen M. Manalo, Maria Jesusa B. Banal-Silao

**Affiliations:** Division of Reproductive Endocrinology and Infertility, Department of Obstetrics and Gynecology, University of the Philippines Manila - Philippine General Hospital, Metro Manila, Philippines

**Keywords:** Fertility preservation, Philippines, Reproductive aging

## Abstract

**Background:**

The demand for fertility preservation has increased substantially over the past decade as more women wish to delay childbearing and with improved survival outcomes of various medical conditions. This study evaluated the awareness and perceptions of Filipino obstetrician-gynecologists on fertility preservation.

**Methods:**

A cross-sectional survey was conducted among diplomates and fellows of the Philippine Obstetrical and Gynecological Society from September to December 2021. A self-administered questionnaire with 24 items was distributed online. Univariate descriptive statistics were reported as means for continuous variables and frequencies with percentage for categorical variables. Differences in responses were tested using the chi-square test.

**Results:**

A total of 215 respondents completed the survey. Majority of the respondents were female, general obstetrician-gynecologists practicing in the National Capital Region. There was an overall positive perception of fertility preservation, with 98.60% agreeing that discussions about childbearing intentions should be initiated. Most participants (98.60%) were aware of fertility preservation but had varying levels of awareness of the different techniques. Fifty-nine percent of the respondents were unaware of regulations on fertility preservation. Setting up dedicated centers for fertility preservation and offering it as a public service were viewed as necessary by the respondents.

**Conclusions:**

This study underscored the need to increase awareness of fertility preservation techniques among Filipino obstetrician-gynecologists. Meeting the need for comprehensive guidelines and centers is essential to promote fertility preservation in the country. Efficient referral systems and multidisciplinary approaches should be established for holistic care.

## Introduction

One of the most critical milestones in reproductive medicine is the advent of fertility preservation. Various fertility preservation techniques allow men and women with compromised fertility a chance to achieve reproductive capacity at a later time. While advances in cancer therapy have led to an increasing number of young patients who survive, a crucial sequela is loss of fertility due to the gonadotoxic profile of current regimens [1]. The field of Oncofertility is a network of different subspecialties focused on techniques to restore reproductive function in patients with malignancies [2]. Aside from cancer patients, fertility preservation has been widely applied to patients with benign conditions such as genetic disorders, autoimmune disorders, and other diseases predisposing to premature gonadal failure. Women who wish to postpone childbearing for social and professional reasons likewise benefit from fertility preservation [3–6].

Age is a critical factor in the Patient-Oriented Strategies Encompassing Individualized Oocyte Number (POSEIDON) for women with poor ovarian response to stimulation [7]. Age directly affects oocyte quality and embryo ploidy. Studies have shown that the number of euploid blastocysts decline after 34 years [8]. Advanced female age and decreased ovarian reserve were shown to be prevalent in POSEIDON patients. This emphasizes the need for counseling on the importance of age and ovarian reserve on the prospects of future fertility [7, 8]. Women at risk of infertility should be identified and provided information specific to their needs. Information regarding the impact of malignancy and other diseases on reproductive function, the effect of treatment on fertility, fertility preservation options, issues relating to cryopreservation storage, infertility and fertility treatments, pregnancy after gonadotoxic treatment, and other childbearing and parenting options should be presented to patients [9].

The Philippine Society for Fertility Preservation was established in 2019, reflecting the growing need and interest to improve and promote its practice. Awareness of established fertility preservation techniques is essential to ensure appropriate counseling of patients and referral to a specialist. Currently available fertility preservation strategies in females include embryo cryopreservation, mature oocyte cryopreservation, ovarian tissue cryopreservation, ovarian suppression with GnRH analogs, ovarian transposition, and fertility-sparing surgeries [9]. Meanwhile, sperm cryopreservation is the only established fertility preservation method in adolescent and adult males [9].

In the Philippines, fertility preservation techniques are only offered in private centers and paid through out-of-pocket expenses. At the time of writing, no government-funded facility offer these procedures. Expenses are not covered by the Philippine Health Insurance Corporation nor by health maintenance organizations.

Despite the rapid progress of fertility preservation in clinical practice, knowledge of its availability is lacking among clinicians [10]. This paucity of knowledge from healthcare providers on the protection of reproductive function certainly affects the patient’s knowledge, attitude, behavior, and perspective. The current study aimed to assess obstetrician-gynecologists awareness and perception of fertility preservation. It was timely and relevant to conduct this study to determine the current status and barriers to improving the practice of fertility preservation in the country. This was the first study among post-residency obstetrician-gynecologists in the Philippines.

## Methods

A cross-sectional survey was conducted among diplomates and fellows of the Philippine Obstetrical and Gynecological Society (POGS) from September to December 2021. A hyperlink to the online survey was sent by electronic mail to the target participants with society’s support and distributed over social media. The minimum required sample was 209 of the 4500 accredited obstetrician-gynecologists in the country. This was computed using the Cochran formula and based on the study by Fritz et al. [11], which reported that 82.8% of obstetricians believe that fertility discussions should be routinely part of the examinations. The sample size was computed with a 5% margin of error and a design effect of 1.0. Non-probability sampling and consecutive enrollment of participants were done until the sample size was achieved.

A self-administered survey patterned from the study of Chung et al. [12]. was utilized. The questionnaire was composed of 24 items divided into two sections. The first section included questions on the demographic profile of the participants. The second section assessed the awareness and perception of fertility preservation. Pilot testing of the questionnaire to 15 subjects was performed before the survey proper.

Univariate descriptive statistics were reported as mean for continuous variables and frequency with percentage for categorical variables. Differences in responses were tested using the chi-square test. A *P value* of < 0.05 was considered statistically significant. All analyses used STATA 14 (Stata Corp Inc).

## Results

A total of 215 participants accomplished the online questionnaire. The mean age of the respondents was 42.98 ± 10.59 years. The majority were female (94.88%), Catholic (80.94%), married (68.37%), and had children (65.12%). The geographical regions were represented, with the National Capital Region (NCR) being the most represented. The sociodemographic data of the respondents are summarized in Table 1.

**Table 1:** Sociodemographic Characteristics of the Study Population

	n	% (N = 215)
**Age**	42.9 ± 10.59	
**Sex**		
Female	204	94.88%
Male	11	5.12%
**Marital Status**		
Married	147	68.37%
Single	63	29.30%
Widowed	2	0.93%
Separated	3	1.30%
**Religion**		
Catholic	174	80.93%
Christian	20	9.30%
Muslim	15	6.98%
Seventh Day Adventist	4	1.86%
Protestant	1	0.47%
Jehovah’s witness	1	0.47%
**With Children**		
Yes	140	65.12%
No	75	34.88%
**Geographic Location**		
BARMM	8	3.72%
CAR	7	3.26%
MIMAROPA	3	1.40%
NCR	81	37.67%
Region I	28	13.02%
Region II	3	1.40%
Region III	18	8.37%
Region IVA	18	8.37%
Region V	2	0.93%
Region VI	6	2.79%
Region VII	9	4.19%
Region VIII	7	3.26%
Region IX	3	1.40%
Region X	5	2.33%
Region XI	11	5.12%
Region XII	4	1.86%
Region XIII	2	0.93%

Most of the participants belonged to private non-university affiliated hospitals (27.91%) and had practiced for one to five years (44.65%). General obstetrician-gynecologists constituted 57.21% of the study population. Of the 42.79% specialists, the most frequently identified specialties were Ultrasound (12.56%) and Reproductive Endocrinology (10.70%). Table 2 provides an occupational summary of the population.

**Table 2:** Occupational Profile of the Study Population

	n	% (N = 215)
**Affiliation**		
Public university-affiliated teaching hospital	47	21.86%
Private university-affiliated teaching hospital	30	13.95%
Public non-university affiliated hospital	45	20.93%
Private non-university affiliated hospital	60	27.91%
Private clinic	33	15.35%
**Years in Practice**		
1–5 years	96	44.65%
6–10 years	37	17.21%
11–15 years	20	9.30%
16–20 years	19	8.84%
>20 years	43	20.00%
**Practice or Specialization**		
General OBGYN	123	57.21%
Ultrasound	27	12.56%
Reproductive Endocrinology	23	10.70%
Maternal-Fetal Medicine	13	6.05%
Gynecologic Oncology	8	3.72%
Trophoblastic Diseases	6	2.79%
Infectious Diseases	5	2.33%
Minimally Invasive Surgery	4	1.86%
Urogynecology	3	1.40%
Family Planning	3	1.40%

Majority of the respondents agreed that obstetrician-gynecologists should initiate discussions with patients about their childbearing intentions (98.60%) and age-related fertility decline (97.67%). Obstetrician-gynecologists largely believed that discussion of natural fertility decline should be part of a well-woman annual examination, with agreement by 96.28%.

Almost all participants (98.60%) were aware of fertility preservation and were familiar with at least one method or procedure. Only 32.56% were familiar with all techniques, including fertility-sparing surgeries, the use of GnRH agonists, sperm freezing, oocyte freezing, embryo freezing, and ovarian or testicular tissue freezing. Most respondents (81.40%) were aware of fertility-sparing surgeries. Approximately half (45.12%) of the participants have not referred patients for fertility preservation in the twelve months before the study proper. Only seven respondents were able to refer patients for all the mentioned procedures (Table 3).

**Table 3:** Awareness of Filipino obstetrician-gynecologists toward fertility preservation

Item	n (%) (N = 215)
Should an OB/GYN initiate discussions with patients about their potential childbearing intentions?	
Yes	212 (98.60%)
No	3 (1.40%)
**Should an OB/GYN initiate discussions about age-related fertility decline with patients?**	
Yes	210 (97.67%)
No	5 (2.33%)
**Should discussing the natural decline in fertility with age be part of a well-woman annual exam with a gynecologist?**	
Yes	207 (96.28%)
No	8 (3.71%)
**Are you aware of fertility preservation?**	
Yes	212 (98.60%)
No	3 (1.40%)
**Are you familiar with the following fertility preservation procedures? Respondents were allowed to choose more than one.**	
Fertility-sparing surgeries	175 (81.40%)
GnRH agonists	130 (60.47%)
Sperm freezing	122 (56.74%)
Oocyte freezing	185 (86.05%)
Embryo freezing	147 (68.37%)
Ovarian or testicular tissue freezing	93 (43.26%)
All of the above	70 (32.56%)
**Have you referred patient(s) for the following fertility preservation procedures over 12 months? Respondents were allowed to choose more than one.**	
Fertility-sparing surgeries	78 (36.28%)
GnRH agonists	42 (19.53%)
Sperm freezing	19 (8.84%)
Oocyte freezing	49 (22.79%)
Embryo freezing	23 (10.70%)
Ovarian or testicular tissue freezing	11 (5.11%)
All of the above	7 (3.26%)
No	97 (45.12%)
**Are you aware of a special clinic or specialists who would be able to accept your referrals for fertility preservation?**	
Yes	187 (86.98%)
No	28 (13.02%)

Respondents were largely aware (86.98%) of a particular clinic or specialist who can accept referrals for fertility preservation. Of note, 28 respondents (13.02%) were unaware of any facility or specialist. In 43.72%, the patient’s desire to have children was identified as the most critical factor when deciding on fertility preservation in medical indications, followed by age (35.81%) and prognosis (10.23%).

Majority of the participants (93.49%) deemed it necessary to set up dedicated centers for fertility preservation. About 91.63% think it should be offered as a public health service. Standard educational materials were deemed essential in enhancing patient understanding of fertility preservation. More than half (59.07%) are unaware of regulations relating to fertility preservation, but 98.14% support establishing guidelines. Three respondents did not wish to know more about fertility preservation (Table 4).

**Table 4:** Perceptions of Filipino obstetrician-gynecologists toward fertility preservation

Item	n (%) (N = 215)
If there are no problems with resources, funding, and technical expertise, which of the following is the single most important factor you think you will consider when deciding on fertility preservation in medical indications?	
Age of the patient	77 (35.81%)
Patient’s desire to have children	94 (43.72%)
Prognosis of patient	22 (10.23%)
Time available before gonadotoxic treatment	9 (4.19%)
Type of cancer	9 (4.19%)
Religion of patient	2 (0.93%)
Marital status of patient	2 (0.93%)
**Is setting up a dedicated clinic/center for fertility preservation counseling necessary?**	
Yes	201 (93.49%)
No	14 (6.51%)
**Do you think fertility preservation should be available as a public service?**	
Yes	197 (91.63%)
No	18 (8.37%)
**Do you think that standard educational materials provided by the professional bodies are important to you for counseling patients to enhance their understanding of fertility preservation?**	
Yes	212 (98.60%)
No	3 (1.40%)
**Have you heard of regulations relating to fertility preservation?**	
Yes	88 (40.93%)
No	127 (59.07%)
**Do you think practice guidelines are required for fertility preservation?**	
Yes	211 (98.14%)
No	4 (1.86%)
**Do you want to know more about fertility preservation?**	
Yes	212 (98.60%)
No	3 (1.40%)

The likelihood of discussing fertility-related practices was not different across characteristics of fellows. However, the analysis is limited by the inadequate number of participants per characteristic category. Cells with a frequency of less than five were merged with other cells to ensure adequacy for analysis.

The likelihood of having an awareness of fertility-related practices was not different across characteristics of fellows except for a few geographic locations and subspecialties. Those in Luzon are 2.26 times more likely to be aware of regulations on fertility preservation than those in the NCR. The Philippines is composed of three major islands known as Luzon, Visayas, and Mindanao. For the analysis, the National Capital Region was separated from Luzon because it houses most of the centers able to provide fertility preservation techniques and has the most number of specialists in the country. Provinces included in Luzon were Regions I, II, III, IV-A, MIMAROPA, V, and CAR. Luzon is generally considered to be more urbanized than provinces in Visayas and Mindanao. Distribution of health infrastructures and human resources is skewed toward Luzon and the National Capital Region.

Respondents with subspecialties other than Reproductive Endocrinology have a 51% reduced odds of having an awareness of these regulations than general obstetrician-gynecologists. Reproductive endocrinologists have 80% reduced odds of agreeing on setting up fertility preservation counseling compared to general obstetricians. On the other hand, Christians have 20% reduced odds of agreeing on the need for practice guidelines than Roman Catholics.

## Discussion

Fertility preservation has continued to gain worldwide attention over the years. A local study conducted by Factor and Novero was the first attempt to examine Filipino practitioners’ knowledge, attitudes, and practices on fertility preservation [13]. The study included 213 surgical oncologists, medical oncologists, and radiation oncologists. Majority of their study participants acknowledged knowing only minimal information. Only 38% have referred patients to fertility specialists, citing lack of knowledge, poor success rates of fertility preservation, poor patient prognosis, and high costs [13].

The current study is the first to describe the awareness and perceptions of Filipino obstetrician-gynecologists about reproductive aging and fertility preservation. The majority of the study respondents were female because they comprise 95% of the diplomates and fellows of the Philippine Obstetrical and Gynecological Society. Being the primary provider of reproductive healthcare, it is reassuring that majority of the respondents agreed that discussions about potential childbearing intentions and age-related fertility decline should be initiated during an annual examination. The International Fertility Decision-Making Study highlighted the lack of knowledge about fertility in 10,045 reproductive-aged men and women in over 79 countries [14]. Counseling increases patient understanding, allows informed decisions about her future reproductive plans, and encourages better patient participation.

There is a high awareness of fertility preservation among the respondents. One of the main objectives of the Philippine Society for Fertility Preservation (PSFP) is to promote the science and practice of fertility preservation. The society conducts regular conferences, meetings, and discussions on scientific information and treatment advances. There were varying levels of awareness of the different techniques. Most were familiar with at least one fertility preservation technique. Meanwhile, only a third of the study population knew all methods. Unawareness may lead to the underutilization of available methods of fertility preservation. This emphasizes the need to educate more obstetrician-gynecologists through fertility preservation awareness campaigns and continuing medical education activities, including seminars and workshops.

Not surprisingly, the highest level of awareness was associated with fertility-sparing surgeries. Fertility-sparing surgery entails preserving at least a portion of an ovary and the uterus. These are limited to early-stage malignancies and include conization or trachelectomy for cervical cancer and unilateral salpingo-oophorectomy for ovarian cancer. Clinicians should provide appropriate information about oncologic and pregnancy outcomes through an individualized patient approach [15]. Obstetrician-gynecologists were likely to be most aware of fertility-sparing surgeries as they perform the surgeries themselves, and specialists provide further treatment.

Despite the high level of awareness, half of the respondents had not referred patients for fertility preservation, and majority desired to know more information. The study’s findings were similar to the reports of Harzif et al. among obstetrician-gynecologists in Indonesia [16]. Identified hindrances were financial constraints, poor success rates of fertility preservation techniques, poor prognosis of patients, and lack of physician knowledge. These underscore that information among obstetrician-gynecologists is lacking. Aside from these, the European Society of Human Reproduction and Embryology (ESHRE) listed limited public awareness of fertility and fertility preservation, limited awareness of oncologists on fertility preservation options, lack of referral pathways, and unavailability of every technique as barriers to access to fertility preservation [9]. Further local studies on the knowledge, attitudes, and practices of Filipino obstetrician-gynecologist may be undertaken to examine the perceived barriers to the provision of much-needed fertility preservation techniques.

Most respondents saw setting up dedicated centers for fertility preservation as necessary. The study shows that reproductive endocrinologists have 80% lower odds of agreeing on this than general obstetrician-gynecologists. A small proportion of the study population was unaware of any facility or specialist. In the Philippines, fertility preservation techniques are mainly performed in reproductive centers offering in vitro fertilization. There are only eight centers and 147 infertility specialists able to provide these services in the country. Access to these centers is available to reproductive endocrinologists, which may explain the decreased support for establishing dedicated facilities. Encompassing help from all specialists should be elicited to promote fertility preservation.

Early referral of women with malignancy at the time of diagnosis and before treatment commencement is the key to maximizing the success of fertility preservation and allows a greater window of opportunity for preserving fertility [12]. As primary doctors of women with gynecologic malignancies, gynecologic oncologists should refer them for reproductive counseling as soon as the diagnosis is made. The ESHRE advocates a model of care for patients eligible for fertility preservation. Central to this model is the awareness of fertility preservation options and the training of healthcare providers. The clinical care team should provide essential information and referrals for fertility preservation consultation. Fertility preservation counseling is provided by specialists after a thorough patient assessment [9]. There is a need for quick and efficient referral systems.

The high cost of most fertility preservation techniques and patient financial constraints have impeded widespread local use. Most respondents agreed that these techniques should be offered as a public health service to mitigate access issues. A multilevel approach is essential to address issues specific to patients and their families, clinicians, organizations, policymakers, and the general population [9].

Fertility preservation is a significant issue in women diagnosed with malignancy. A survey of young women undergoing therapy showed that childbearing remains a priority [17]. Diminished reproductive capacity and fertility loss are leading causes of anxiety and depression among this population. Studies suggest that the risk of infertility has a significant impact on the decision-making process of young cancer patients [18]. In a prospective cohort study among 425 women with newly diagnosed breast cancer, 1% decided not to receive chemotherapy, 2% chose one chemotherapy regimen over another, 1% considered not receiving endocrine therapy, 3% chose not to receive endocrine therapy, and 11% considered receiving endocrine therapy for five years due to concerns in fertility [19]. Similarly, the study respondents deemed a patient’s desire to have children the most important factor when deciding on fertility preservation in medical conditions. It is, therefore, worthwhile to investigate patient perceptions and access to the different techniques in the local setting. Patient age and prognosis were among the top considerations. These again stress the need for timely counseling.

Comprehensive recommendations and clinical guidelines on fertility preservation should be established and communicated. ESHRE published its first evidence-based guideline on female fertility preservation for healthcare professionals in 2020 [9]. Socio-economic factors relating to the respondents’ place of practice and affiliation could influence their knowledge, attitudes, and practices on fertility preservation. Private practitioners manage a different subset of patients compared to those in public facilities. Their patients are better able to afford fertility preservation techniques. As such, they are more exposed and knowledgeable on fertility preservation. Considering the current laws, patient population, and socioeconomic factors, these guidelines need to be optimized in the local setting. Interestingly, practitioners in Luzon were 2.26 times more likely to be aware of regulations on fertility preservation than those in the NCR. Subgroup analysis of participants in Luzon showed that the majority have been practicing for one to five years, while most of those in NCR has been practicing for more than 16 years. This may be due to more active personal inquiry by younger clinicians or better participation in regional campaigns. The availability of fertility preservation techniques in the NCR should be an impetus for practitioners in this area to improve awareness. Subspecialties other than reproductive endocrinology had 51% reduced odds of awareness of existing guidelines compared to general obstetrician-gynecologists. Their specialized practices may deter them from acquiring further information in this growing field. As primary reproductive healthcare providers, all obstetrician-gynecologists should be knowledgeable about recommendations and guidelines.

Overall, Filipino obstetrician-gynecologists have an encouraging positive perception of fertility preservation. There is a need for further education on the locally available techniques. The information presented by this study can be applied in the framework of establishing local guidelines and designing a curriculum for training. A multidisciplinary team with reproductive specialists, an insurance coverage system, comprehensive laws, and practice guidelines should be prioritized.

## Conclusion

Reproductive aging and fertility preservation are emerging fields in managing reproductive-aged women. This is the first local study that evaluated the awareness and perceptions of post-residency obstetrician-gynecologists on fertility preservation. The study showed a reassuring positive perception of fertility preservation but a gap in the awareness of different approved methods. A multidisciplinary approach and dedicated facilities should be established for fertility consultation, risk assessment, and counseling. Healthcare delivery should be organized to meet the increasing need for fertility preservation.

## Limitations of the study

The study employed non-probability sampling and consecutive enrolment of participants until the sample size was met. Selection and response bias may have influenced the results of the study. Participation in a self-directed online questionnaire entails the awareness of the sample to the existence of the survey. The number of physicians who actually received the survey is uncertain. This was minimized by distribution of the questionnaire by the Philippine Obstetrical and Gynecological Society to its registered members. Regular posting of the survey to various social media platforms was also conducted to improve visibility and response. To minimize response bias, the period of data collection was extended after the minimum sample size was met. The number of Gynecologic Oncology specialists who completed the survey was only eight due to the sampling method employed. The study did not assess the specific reasons for non-referral for fertility preservation techniques.

## Recommendations

Further studies with the recruitment of gynecologic oncologists may be undertaken. Another vital area of research is the investigation of perceived barriers to the provision of timely and appropriate fertility preservation techniques. The knowledge and perceptions of patients on fertility preservation should also be investigated.

## Data Availability

The datasets used and/or analyzed during the current study are available from the corresponding author on reasonable request.
